# The Development of Machine Learning-Assisted Software for Predicting the Interaction Behaviours of Lactic Acid Bacteria and *Listeria monocytogenes*

**DOI:** 10.3390/life15020244

**Published:** 2025-02-06

**Authors:** Fatih Tarlak, Jean Carlos Correia Peres Costa, Ozgun Yucel

**Affiliations:** 1 Department of Bioengineering, Faculty of Engineering, Gebze Technical University, Gebze 41400, Kocaeli, Turkey; 2 Applied Mycology Unit, Department of Food Technology, Engineering and Science, AGROTECNIO-CERCA Center, University of Lleida, 25198 Lleida, Spain; jeancarlos.correia@udl.cat; 3 Department of Chemical Engineering, Gebze Technical University, Gebze 41400, Kocaeli, Turkey

**Keywords:** biopreservation, interaction model, machine learning, lactic acid bacteria, *Listeria monocytogenes*

## Abstract

Biopreservation technology has emerged as a promising approach to enhance food safety and extend shelf life by leveraging the antimicrobial properties of beneficial microorganisms. This study aims to develop precise predictive models to characterize the growth and interaction dynamics of lactic acid bacteria (LAB) and *Listeria monocytogenes*, which serve as bioprotective agents in food systems. Using both traditional and machine learning modelling approaches, we analyzed data from previously published growth curves in broth (BHI) and milk under isothermal conditions (4, 10, and 30 °C). The models evaluated mono-culture conditions for *L. monocytogenes* and LAB, as well as their competitive interactions in co-culture scenarios. The modified Gompertz model demonstrated the best performance for mono-culture simulations, while a combination of the modified Gompertz and Lotka–Volterra models effectively described co-culture interactions, achieving high adjusted R-squared values (*adjusted R*^2^ = 0.978 and 0.962) and low root mean square errors (*RMSE* = 0.324 and 0.507) for BHI and milk, respectively. Machine learning approaches further validated these findings, with improved statistical indices (adjusted *R*^2^ = 0.988 and 0.966, *RMSE* = 0.242 and 0.475 for BHI and milk, respectively), suggesting their potential as robust alternatives to traditional methods. The integration of machine learning-assisted software developed in this work into predictive microbiology demonstrates significant advancements by bypassing the conventional primary and secondary modelling steps, enabling a streamlined, precise characterization of microbial interactions in food products.

## 1. Introduction

In recent years, consumer awareness about foodborne illnesses and the risks linked to the excessive use of chemical preservatives in food products has significantly increased. This growing concern has pushed the agriculture and food industries toward adopting innovative technologies that prioritize food safety and quality while minimizing dependency on synthetic additives [1]. The European Rapid Alert System for Food and Feed (RASFF) reported 122 notifications of *Listeria monocytogenes* in the milk and milk products category over the past five years. Alarmingly, in just the first month of 2025, RASFF recorded 5 notifications of this pathogen in products like cheese, highlighting the persistent challenge it poses to food safety. One promising solution to this issue is biopreservation, which employs microorganisms, often classified as “Generally Recognized as Safe” (GRAS), and/or their metabolites to inhibit undesirable microorganisms. This approach maintains an ecological balance by suppressing the growth of pathogens and spoilage agents [2]. By adding natural microbiota or antagonist microorganisms and their metabolites, biopreservation extends the shelf life of food products while offering a health- and environmentally friendly alternative to conventional preservatives. This aligns with consumer demands for sustainable and natural solutions, ensuring food safety without compromising product quality [3,4,5,6].

Lactic acid bacteria (LAB) represent a diverse group of microorganisms extensively used in food production as microbial food cultures (MFCs) [7,8]. Despite the absence of specific regulations governing LAB as MFCs in the European Union, their longstanding history of safe use ensures they are classified as food additives and legally permissible without prior regulatory approval [3,9]. LAB are Gram-positive, non-sporulating, anaerobic, or facultatively aerobic bacilli and cocci. This group includes genera such as *Latilactobacillus*, *Carnobacterium*, *Enterococcus*, *Lactococcus*, *Leuconostoc*, *Pediococcus*, and *Streptococcus*, all of which share a common metabolic trait: the fermentation of carbohydrates with lactic acid as the primary product [10]. These metabolic capabilities underpin their critical role in food fermentation and preservation, making LAB indispensable to the food industry.

LAB play a pivotal role in the food industry, functioning both as starter cultures in fermentation processes and as bioprotective cultures in biopreservation strategies [11,12,13]. Their efficacy is rooted in their safe metabolic activities; as LAB proliferate in food matrices, they metabolize available sugars to produce organic acids and a variety of antimicrobial metabolites. This metabolic activity is integral to biopreservation, enhancing the microbial safety of foods while also contributing to sensory, technological, nutritional, and health-related benefits [14]. LAB are recognized as safe by the United States Food and Drug Administration [15] and meet the Qualified Presumption of Safety (QPS) criteria set by the European Food Safety Authority [16], reaffirming their suitability for use as bioprotective cultures in diverse food applications.

Predictive microbiology has developed into a specialized scientific field dedicated to using mathematical models and computational methods to predict the growth, survival, and dynamics of microorganisms in food products and other environments [17]. These predictive tools empower scientists, food industry professionals, and regulatory authorities to assess microbial spoilage and contamination risks effectively. By employing these predictive tools, stakeholders can make informed decisions to enhance food safety, optimize storage conditions, and extend product shelf life. The primary objective of predictive microbiology is to strengthen food safety protocols by accurately forecasting microbial behaviour under various environmental conditions, thereby ensuring consumer health and safety while maintaining food quality.

In predictive microbiology, mathematical frameworks are typically categorized into three types: primary, secondary, and tertiary models. Primary models describe microbial growth dynamics over time under static environmental conditions using mathematical equations. Secondary models, on the other hand, quantify the influence of intrinsic and extrinsic factors—such as temperature, oxygen levels, pH, and water activity (a_w_)—on the kinetic parameters derived from primary models. Temperature is a key factor influencing microbial growth patterns in food products, with the Ratkowsky and Arrhenius models widely used to evaluate microbial responses to temperature changes. Tertiary models combine the outputs of primary and secondary models with computational tools, enabling advanced predictive analysis and simulation through specialized software V01 [18]. These integrated models serve as powerful tools for enhancing food safety and optimizing storage conditions.

Food environments represent complex microbial ecosystems where diverse microbial communities coexist, interact, and influence the presence and behaviour of pathogens [13]. These interactions involve environmental recognition and the exchange of molecular and genetic information, leading to outcomes that can be positive, negative, or neutral. The intricate nature of these interactions is influenced by the microbial species involved, their modes of interaction, and the factors used in food preservation, making it challenging to fully understand their dynamics. Microbial interactions in food can be non-specific, relying on physical contact or quorum-sensing mechanisms, often driven by competition for resources. Alternatively, interactions can be specific, occurring without physical contact and involving the production of antimicrobial metabolites such as bacteriocins and organic acids, which alter the growth environment [3]. Although these mechanisms are increasingly understood, much of the research on microbial interactions in food systems remains qualitative, highlighting the need for more quantitative studies to unravel their complexities [19].

The primary objective of this research is to develop a machine learning-based approach as an innovative alternative to traditional modelling techniques, aiming to accurately predict the behaviour of a critical food safety pathogen in co-culture with other microorganisms. The necessity for developing machine learning-assisted software arises from the limitations of conventional methods, which often involve labour-intensive primary and secondary modelling steps and may not fully capture the complexity of microbial interactions in food environments. This study represents the first effort to integrate machine learning into predictive microbiology specifically for modelling these complex microbial interactions. By streamlining the predictive modelling process and enhancing accuracy, the machine learning-assisted software offers a standardized and efficient tool that can significantly advance food safety management and microbiological risk assessment. This innovative approach not only improves the prediction of microbial behaviour but also addresses the need for more robust, data-driven methodologies in the field.

## 2. Material and Methods

### 2.1. Study Structure

**Figure 1:**
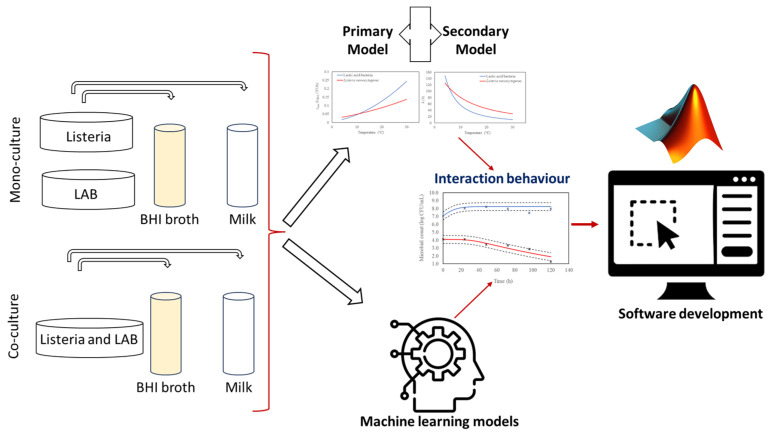
Overview of the steps followed in this study, outlining the key stages of the methodology and data analysis approach.

The study was conducted in four key phases. The first phase involved collecting growth data for LAB and *L. monocytogenes* in both broth and milk environments under various isothermal conditions (4, 10, and 30 °C). In the second phase, a combined modelling approach was employed, integrating several primary models—modified Gompertz, logistic, Baranyi, and Huang—with the secondary Ratkowsky model to define and analyze growth kinetics. The third phase focused on modelling and simulating the interactions between LAB and *L. monocytogenes* in broth and milk environments, using both traditional modelling techniques and machine learning methodologies to capture microbial dynamics. The final phase centred on the development of specialized software designed to facilitate comparisons between conventional modelling approaches and machine learning-based methods. A comprehensive flowchart (Figure 1) provides a visual summary of these phases, with detailed explanations outlined in the following sections.

### 2.2. Data Collection

Bacterial growth data for a cocktail of three LAB strains—*Lactococcus lactis* ssp. *lactis*, strain 340; *L. lactis* ssp. *lactis*, strain 16; and *Lacticaseibacillus casei* ssp. *casei*, strain 208—and *L. monocytogenes* ATCC 7644 were sourced from the study by Francesca Iulietto et al. [20]. This research provided detailed methodologies for monitoring these bacteria in brain heart infusion broth (BHI) and skim milk under isothermal conditions at 4, 10, and 30 °C. Growth curves were analyzed across three scenarios: LAB formulations alone, LAB in competition with *L. monocytogenes*, and *L. monocytogenes* in mono-culture. The experiments began by growing freeze-dried *L. monocytogenes* ATCC 7644 in brain heart infusion (BHI) broth (BD Difco, Franklin Lakes, NJ, USA) at 30 °C for 48 h to obtain an initial concentration of approximately 8 log CFU/mL. Purity was verified by spreading the culture on Oxford agar (Listeria selective agar, Oxoid, UK) supplemented with Listeria selective supplement and incubating at 37 °C for 48 h.

For the subsequent trials, two main media were used: skim milk (BD Difco, 232100) and BHI broth (BD Difco). A LAB formulation—comprising *Lactococcus* and *Lacticaseibacillus* spp. along with *E. faecium* UBEF-41 in a 2:1:1 ratio—was inoculated into 200 mL of either skim milk or BHI at an initial concentration of about 7 log CFU/mL. Then, *L. monocytogenes* was introduced into the same media at an initial concentration of approximately 4 log CFU/mL.

Growth curves were monitored under three temperature regimes (30, 10, and 4 °C) using an incubator (Sanyo MIR-153, Zevenhuizen, The Netherlands), with continuous temperature verification via a data logger. Bacterial counts for both the LAB formulation and *L. monocytogenes* were recorded at designated intervals: on days 0, 1, 2, 3, 4, and 5 at 30 °C; and on days 0, 2, 7, 9, 14, 18, 21, and 28 at 10 °C and 4 °C. Microbiological analyses included serial dilutions and plating on appropriate selective media (e.g., Oxford agar for *L. monocytogenes*) with incubation conditions tailored to each bacterial species [20]. These data formed the basis for the subsequent modelling and analysis presented in this study.

### 2.3. Primary Models

The modified Gompertz, logistic, Baranyi, and Huang models are frequently used sigmoid functions for modelling bacterial growth patterns. The modified Gompertz model is represented by Equation (1) and the logistic model by Equation (2), each defined under steady environmental conditions [21].

(1) $${x\left(t\right)=x_{0}+(x}_{max}-x_{0}).exp\left\{-exp\left[\frac{r_{max}.e}{{(x}_{max}-x_{0})}.\left(\lambda -t\right)+1\right]\right\}$$

(2) $$x\left(t\right)=x_{0}+\frac{{(x}_{max}-x_{0})}{\left\{1+\exp\left[\frac{{4.r}_{max}}{{(x}_{max\;}-x_{0})}.\left(\lambda -t\right)+2\right]\right\}}$$

where *t* is the time (h), *x*(*t*) is the bacterial population concentration (log CFU/mL) at time *t*, *x*_0_ is the initial bacterial population concentration (log CFU/mL), *x_max_* is the maximum bacterial population concentration (log CFU/mL), *r_max_* is the maximum bacterial growth rate (log CFU/h), and *λ* is the lag phase duration (h).

The Baranyi and Huang models are also popular primary functions for bacterial growth, described by Equations (3) and (4), respectively [22,23].

(3) $$y\left(t\right)=y_{0}+\mu_{max}\cdot F\left(t\right)-\ln\left(1+\frac{e^{\mu_{max}\cdot F\left(t\right)}-1}{e^{\left(y_{max}-y_{0}\right)}}\right)$$

(4) $$y\left(t\right)=y_{0}+y_{max}-ln(e^{y_{0}}+\left[e^{y_{max}}-e^{y_{0}}\right].e^{{-\mu }_{max\cdot }B(t)})$$

where *t* is the time (h), *y*(*t*) is the bacterial population concentration (ln CFU/mL) at time *t*, *y*_0_ is the initial bacterial population concentration (ln CFU/mL), *y_max_* is the maximum bacterial population concentration (ln CFU/mL), *µ_max_* is the maximum specific bacterial growth rate (1/h), *λ* is the lag phase duration (h), and *F*(*t*) and *B*(*t*) are adjustment functions specific to the models.

Due to the different scales used by the main models for quantifying microbial populations, the growth rate values (*r_max_*) from the modified Gompertz, and logistic models are converted into maximum specific growth rate values (*µ_max_*) via multiplication by the natural logarithm (ln) after the fitting process [24].

### 2.4. Secondary Models

Secondary models detail how various environmental factors, like water activity, acidity, and temperature, influence the parameters of primary models [24]. Typically employed after primary model growth data fitting, these models include the Ratkowsky model, which elucidates the relationship between temperature and the maximum specific growth rate [25].

(5) $${\mu_{max}=b}_{1}{\left(T-T_{0}\right)}^{2}$$

where T is the storage temperature (°C), T_0_ is the theoretical lowest bacterial growth temperature (°C), *µ_max_* is the maximum specific growth rate (1/h), and b_1_ is the regression coefficient.

Additionally, *λ* was correlated with *µ_max_* with respect to temperature using Equation (6) [18].

(6) $$\lambda =\frac{b_{2}}{\mu_{max}(T)\;\;}$$

where b_2_ is the regression coefficient, and *µ_max_* (*T*) is a function of temperature.

### 2.5. Interaction Models

#### 2.5.1. Modified Jameson Model

In this study, a modification of the Jameson effect model was used, represented by Equations (7) and (11). This modification includes the parameters *N_criLAB_* and *N_criLm_* that describe the maximum critical concentration that a population should reach to inhibit the growth of the other population [26,27,28].

(7) $$\frac{dN_{LAB}}{dt}=N_{LAB}\cdot \mu_{maxLAB}\cdot \left(1-\frac{N_{LAB}}{N_{maxLAB}}\right)\cdot \left(1-\frac{N_{Lm}}{N_{criLm}}\right)\cdot \left(\frac{Q_{LAB}}{1+Q_{LAB}}\right)$$

(8) $$\frac{dN_{Lm}}{dt}=N_{Lm}\cdot \mu_{maxLm}\cdot \left(1-\frac{N_{Lm}}{N_{maxLm}}\right)\cdot \left(1-\frac{N_{LAB}}{N_{criLAB}}\right)\cdot \left(\frac{Q_{Lm}}{1+Q_{Lm}}\right)$$

(9) $$\frac{dQ_{LAB}}{dt}=Q_{LAB-1}\cdot \mu_{maxLAB}$$

(10) $$\frac{dQ_{Lm}}{dt}=Q_{Lm-1}\cdot \mu_{maxLm}$$

where *N* is the cell concentration (CFU/mL) at time *t*, *μ_max_* is the maximum specific growth rate (h^−1^), *N_max_* is the maximum population density (CFU/mL), *Q* is a measure of the physiological state of cells at time *t*, for lactic acid bacteria (LAB) or *L. monocytogenes* (Lm), and *N_cri_* is the maximum critical concentration (CFU/mL) of lactic acid bacteria (LAB) on *L. monocytogenes* (Lm) and vice versa.

The value of *Q* at *t* = 0 (*Q*_0_) for LAB and *L. monocytogenes* was calculated using Equation (11).

(11) $$Q_{0}=\frac{1}{e^{\left(\mu_{max}\cdot \lambda \right)}-1}$$

#### 2.5.2. Lotka–Volterra Model

The Lotka–Volterra model, also referred to as the predator–prey model, was used according to Equations (12) and (15). This model includes two empirical parameters reflecting the degree of interaction between microbial species (*F_LABLm_* and *F_LmLAB_*) [3,29,30]. Depending on the empirical parameter value for lactic acid bacteria (*F_LABLm_*), the growth of *L. monocytogenes* can be affected in three different ways:

(1) If 0 < *F_LABLm_* < 1, *L. monocytogenes* grows with reduced *µ_max_* after lactic acid bacteria reaches *N_max_*.

(2) If *F_LABLm_* = 1, *L. monocytogenes* stops growing when lactic acid bacteria reach their *N_max_*.

(3) If *F_LABLm_* > 1, the *L. monocytogenes* population declines when lactic acid bacteria reach their *N_max_*.

(12) $$\frac{dN_{LAB}}{dt}=N_{LAB}\cdot \mu_{maxLAB}\cdot \left(1-\frac{N_{LAB}+F_{LABLm}\cdot N_{Lm}}{N_{maxLAB}}\right)\cdot \left(\frac{Q_{LAB}}{1+Q_{LAB}}\right)$$

(13) $$\frac{dN_{Lm}}{dt}=N_{Lm}\cdot \mu_{maxLm}\cdot \left(1-\frac{N_{Lm}+F_{LmLAB}\cdot N_{LAB}}{N_{maxLm}}\right)\cdot \left(\frac{Q_{Lm}}{1+Q_{Lm}}\right)$$

(14) $$\frac{dQ_{LAB}}{dt}=Q_{LAB-1}\cdot \mu_{maxLAB}$$

(15) $$\frac{dQ_{Lm}}{dt}=Q_{Lm-1}\cdot \mu_{maxLm}$$

where *F_LABLm_* and *F_LmLAB_* are, respectively, the competition factor parameters of lactic acid bacteria on *L. monocytogenes* and vice versa. The other parameters are as indicated in Equations (7)–(11).

#### 2.5.3. Machine Learning Model

This study assessed bacterial counts of LAB and *L. monocytogenes* in broth and milk environments, with measurements expressed as log CFU/mL. The dataset included numerical variables (time and temperature) and categorical variables (presence of interactions and type of growth medium). Baseline bacterial population counts at 0 h were used as a reference for analysis. Data preprocessing was performed using Matlab 8.3.0.532 (R2014a) from MathWorks Inc., Natick, MA, USA. Two machine learning methods, Random Forest Regression (RFR) and Gaussian Process Regression (GPR), were applied to analyze the interaction patterns of LAB and *L. monocytogenes*.

RFR constructs predictive models by combining the outputs of multiple decision trees applied to the dataset. This method effectively captures non-linear relationships and adapts well to unfamiliar datasets. By utilizing an ensemble of trees rather than a single tree, RFR minimizes the risk of overfitting and facilitates the analysis of complex predictor-response relationships. It is particularly robust against outliers and demonstrates excellent generalization capabilities, making it well suited for microbial interaction studies.

GPR is a probabilistic, non-parametric Bayesian framework that generates objective functions by creating normal distributions within an infinitely dimensional space. It measures the distances between the estimated output probability density functions, ensuring reliable predictions even in areas with limited data coverage. However, GPR’s reliance on the entire dataset for each prediction introduces significant computational demands, requiring substantial resources for effective implementation [31].

### 2.6. Comparison of the Models

Comparison of models regarding how well they can describe the observed growth data was carried out with statistical indices such as root mean square error (*RMSE*) and adjusted coefficient of determination (*adjusted R*^2^) values given by Equations (16) and (17), respectively:

(16) $$RMSE=\sqrt{\sum_{i=1}^{n}\frac{{\left(x_{obs}-x_{fit}\right)}^{2}}{n-s}}$$

(17) $$adjusted\;R^{2}-\left(\frac{n-1}{n-s}\right)\left(\frac{SSE}{SST}\right)$$

where *x_obs_* is the value obtained in experiments, *x_fit_* is the fitted value, n is the number of observations, s is the number of parameters of the model, *SSE* is the sum of squared residuals, and *SST* is the total sum of squares.

## 3. Results and Discussion

The traditional modelling approach, incorporating the modified Gompertz, logistic, Baranyi, and Huang models, was developed to simulate the growth dynamics of LAB and *L. monocytogenes* in broth (BHI) and milk under isothermal conditions (4, 10, and 30 °C). This approach utilized growth and challenge data, as summarized in Table 1, Table 2 and Table 3, to characterize microbial growth behaviour and interaction patterns.

At 4 °C, *L. monocytogenes* exhibited steady growth when cultivated alone, reaching 8.84 log CFU/mL in BHI and 7.63 log CFU/mL in milk by hour 672. In contrast, under co-culture conditions at 4 °C, significant population decreases were noted, with reductions of up to 3.42 log CFU/mL in BHI and 6.15 log CFU/mL in milk by hour 672. At 10 °C, *L. monocytogenes* multiplied rapidly in mono-culture, peaking at 8.94 log CFU/mL in BHI and 8.09 log CFU/mL in milk. However, in co-culture at 10 °C, the bacterium’s numbers declined by 3.75 log CFU/mL in BHI and 1.72 log CFU/mL in milk over the same period (hour 672). At 30 °C, the protective effect of LAB was particularly pronounced, reducing *L. monocytogenes* to 1.30 log CFU/mL in BHI by hour 120 and completely eliminating it from milk by day 3 (Table 1, Table 2 and Table 3).

The irregularities observed in the growth curves of LAB and *L. monocytogenes* largely stem from indirect interactions driven by LAB’s metabolic activities. LAB produce bacteriocins—such as nisin, sakacin, and plantaricin—that specifically target pathogens like *L. monocytogenes*, and these compounds can remain active even when produced in separate cultures. Consequently, studies have shown that residual bacteriocins significantly reduce Listeria counts in various food matrices, including fresh cheese and raw sausage [32]. Moreover, LAB lower the pH of their environment through lactic acid production, creating conditions less favourable for *Listeria* growth [33]. Notably, these antimicrobial effects are not confined to co-culture scenarios; bacteriocins and organic acids can persist in the medium, leading to residual inhibition even in mono-cultures. In addition, factors such as temperature and medium composition further influence *Listeria*’s growth dynamics, contributing to the observed variability and irregularity in both co-culture and mono-culture settings.

Each primary model demonstrated strong capabilities in simulating microbial behaviour across the tested conditions. Statistical metrics indicated that the modified Gompertz model consistently outperformed other primary models in both BHI and milk, as evidenced by lower RMSE and higher adjusted R^2^ values (Table 4, Table 5 and Table 6). For LAB growth in BHI, the highest RMSE was 0.276, and the lowest adjusted R^2^ was 0.781, while for *L. monocytogenes*, the corresponding values were 0.505 and 0.925. In milk, the LAB growth model exhibited a maximum RMSE of 0.230 and a minimum adjusted R^2^ of 0.925, whereas *L. monocytogenes* growth predictions showed a maximum RMSE of 0.429 and a minimum adjusted R^2^ of 0.916.

**Figure 2:**
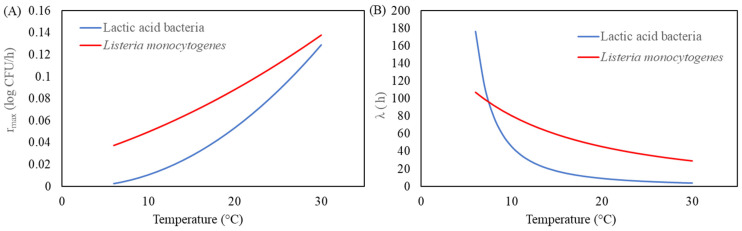
Relationship between temperature and (**A**) *r_max_* and (**B**) *λ* using modified Gompertz model and secondary model of Ratkowsky for brain heart infusion broth. The solid blue and red lines represent the predicted for lactic acid bacteria (LAB) and *L. monocytogenes*, respectively.

**Figure 3:**
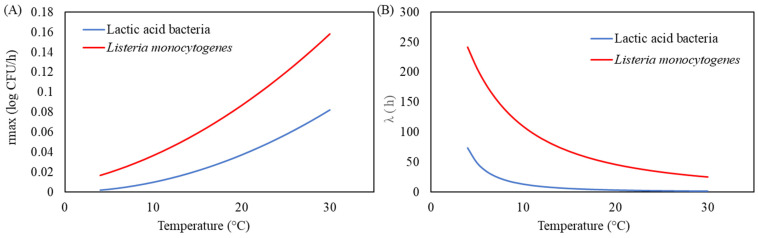
Relationship between temperature and (**A**) *r_max_* and (**B**) *λ* using modified Gompertz model and secondary model of Ratkowsky for milk. The solid blue and red lines represent the predicted for lactic acid bacteria (LAB) and *L. monocytogenes*, respectively.

The modified Gompertz model was selected as the superior primary model due to its robustness in capturing the growth dynamics of both microorganisms. Key parameters, including the maximum growth rate (*µ_max_*) and lag phase duration (*λ*), provided critical insights into microbial behaviour. With increasing storage temperatures from 4 °C to 30 °C, *µ_max_* for LAB and *L. monocytogenes* increased significantly, while *λ* decreased, indicating faster growth rates and shorter lag phases at higher temperatures (Figure 2 and Figure 3).

**Figure 4:**
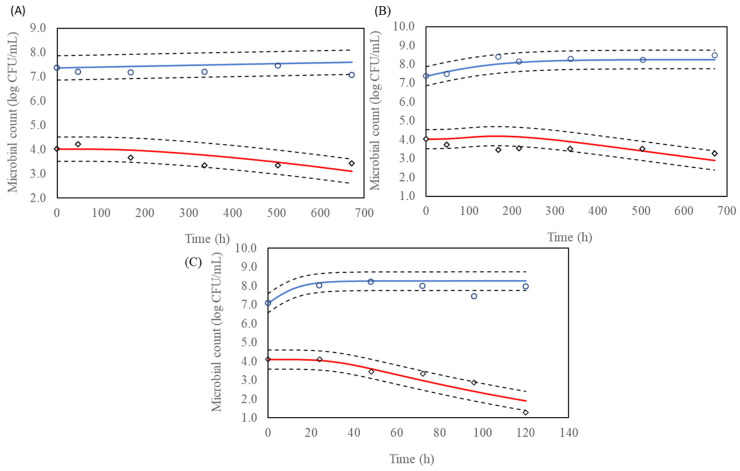
Microbial counts of lactic acid bacteria (LAB) (○) and *L. monocytogenes* (◊) in co-culture using brain heart infusion (BHI) broth at (**A**) 4 °C, (**B**) 10 °C and (**C**) 30 °C. The solid blue and red lines represent the predicted growth curves for LAB and *L. monocytogenes*, respectively, based on the Lotka-Volterra model. The dashed lines indicate confidence bands of 95%, used to compare observed versus predicted interaction between LAB and *L. monocytogenes*.

**Figure 5:**
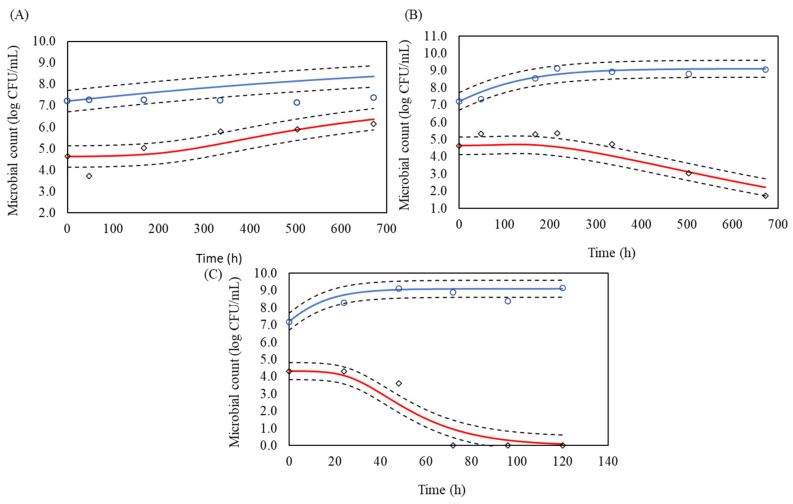
Microbial counts of lactic acid bacteria (LAB) (○) and *L. monocytogenes* (◊) in co-culture using milk at (**A**) 4 °C, (**B**) 10 °C and (**C**) 30 °C. The solid blue and red lines represent the predicted growth curves for LAB and *L. monocytogenes*, respectively, based on the Lotka-Volterra model. The dashed lines indicate confidence bands of 95%, used to compare observed versus predicted interaction between LAB and *L. monocytogenes*.

For interaction modelling, the Lotka–Volterra model outperformed the modified Jameson model in both BHI and milk environments. In BHI, the Lotka–Volterra model achieved an RMSE of 0.324 and an adjusted R^2^ of 0.978, compared to the modified Jameson model’s RMSE of 0.368 and adjusted R^2^ of 0.972. Similarly, in milk, the Lotka–Volterra model recorded superior metrics, with an RMSE of 0.507 and adjusted R^2^ of 0.962, as opposed to the modified Jameson model’s RMSE of 0.563 and adjusted R^2^ of 0.958 (Figure 4 and Figure 5). These results demonstrate the Lotka–Volterra model’s superior ability to capture the complex dynamics of microbial interactions.

Temperature played a significant role in modulating the bioprotective effects of LAB against *L. monocytogenes*. As the storage temperature increased from 4 °C to 30 °C, LAB’s bioprotective effect, quantified using the *F_LABLm_* metric, showed a marked enhancement. This adaptive response underscores LAB’s potential as a natural bioprotective agent, particularly in warmer storage conditions, where its inhibitory effects on *L. monocytogenes* were most pronounced (Table 6).

The integration of machine learning methods, including Random Forest Regression (RFR) and Gaussian Process Regression (GPR), provided an advanced framework for modelling microbial interactions. RFR demonstrated robustness in identifying complex, non-linear relationships within the dataset, while GPR offered high accuracy through its probabilistic Bayesian framework. GPR achieved superior prediction accuracy, with a minimum adjusted R^2^ of 0.966 and a maximum RMSE of 0.475, outperforming traditional models in predicting microbial interactions (Supplementary Figure S1).

The development of a machine learning-assisted software tool was a critical outcome of this study. This tool effectively integrates data-driven machine learning models with traditional microbiological modelling frameworks, providing a user-friendly platform for predicting microbial interactions. Available on the GitHub platform (https://github.com/ftarlak/Interaction-behaviour-predictor, accessed on 7 April 2024), the software bridges the gap between conventional modelling techniques and emerging computational approaches. By enabling accurate simulations without the need for labour-intensive modelling steps, the software offers significant advancements in predictive microbiology.

This study highlights the potential of combining traditional and machine learning approaches to advance predictive microbiology. The superior performance of the modified Gompertz and Lotka–Volterra models, coupled with the accuracy of machine learning techniques, underscores the value of integrating these methodologies. The developed software provides a scalable and efficient solution for modelling microbial growth and interactions, making it a valuable tool for enhancing food safety and preservation strategies. Moreover, the study’s findings emphasize the role of temperature and nutrient composition in shaping microbial behaviour, offering practical insights for food industry applications. The integration of machine learning not only enhances prediction accuracy but also streamlines the modelling process, establishing a robust foundation for future research in predictive microbiology.

## 4. Conclusions

This study demonstrated the effectiveness of both traditional and machine learning approaches in modelling the growth and interactions of LAB and *L. monocytogenes* in BHI and milk under different isothermal conditions. The Lotka–Volterra model outperformed other traditional methods, while Gaussian Process Regression proved to be the most accurate machine learning approach, highlighting its potential to streamline and enhance predictive microbiology. Additionally, the development of a machine learning-assisted software tool provides an accessible and practical solution for food safety management and biopreservation strategy development. This research underscores the transformative role of machine learning in predictive microbiology, paving the way for more efficient and accurate modelling of microbial interactions, ultimately contributing to safer and longer-lasting food products.

## Figures and Tables

**Table 1:** The concentration (log CFU/mL) of lactic acid bacteria (LAB) and *L. monocytogenes* in mono-culture and co-culture set-ups using brain heart infusion broth and milk at 4 °C.

Time (h)	BHI				MILK			
	Mono-Culture		Co-Culture		Mono-Culture		Co-Culture	
	LAB	*L. monocytogenes*	LAB	*L. monocytogenes*	LAB	*L. monocytogenes*	LAB	*L. monocytogenes*
0	7.07 ± 0.03	4.47 ± 0.19	7.37 ± 0.09	4.02 ± 0.34	7.16 ± 0.11	4.66 ± 0.01	7.21 ± 0.06	4.64 ± 0.12
48	7.17 ± 0.10	4.60 ± 0.20	7.19 ± 0.07	4.21 ± 0.27	7.28 ± 0.11	4.65 ± 0.17	7.26 ± 0.09	3.73 ± 0.04
168	7.38 ± 0.06	5.96 ± 0.07	7.17 ± 0.23	3.67 ± 0.06	7.20 ± 0.14	5.33 ± 0.07	7.27 ± 0.04	5.03 ± 0.05
336	7.29 ± 0.11	7.31 ± 0.05	7.20 ± 0.10	3.35 ± 0.05	7.37 ± 0.06	6.24 ± 0.11	7.24 ± 0.08	5.79 ± 0.11
504	7.61 ± 0.06	8.98 ± 0.08	7.45 ± 0.20	3.34 ± 0.03	8.24 ± 0.12	7.09 ± 0.09	7.15 ± 0.15	5.90 ± 0.11
672	7.06 ± 0.14	8.84 ± 0.29	7.06 ± 0.22	3.42 ± 0.28	7.69 ± 0.20	7.63 ± 0.17	7.36 ± 0.10	6.15 ± 0.13

**Table 2:** The concentration (log CFU/mL) of lactic acid bacteria (LAB) and *L. monocytogenes* in mono-culture and co-culture set-ups using brain heart infusion broth and milk at 10 °C.

Time (h)	BHI				MILK			
	Mono-Culture		Co-Culture		Mono-Culture		Co-Culture	
	LAB	*L. monocytogenes*	LAB	*L. monocytogenes*	LAB	*L. monocytogenes*	LAB	*L. monocytogenes*
0	7.07 ± 0.03	4.47 ± 0.19	7.37 ± 0.09	4.02 ± 0.34	7.16 ± 0.11	4.66 ± 0.01	7.21 ± 0.06	4.64 ± 0.12
48	6.85 ± 0.43	5.81 ± 0.16	7.48 ± 0.09	3.73 ± 0.04	7.55 ± 0.07	5.68 ± 0.08	7.34 ± 0.02	5.33 ± 0.16
168	8.39 ± 0.01	8.64 ± 0.04	8.41 ± 0.09	3.45 ± 0.08	8.64 ± 0.09	7.43 ± 0.13	8.56 ± 0.21	5.31 ± 0.05
216	8.24 ± 0.12	8.94 ± 0.14	8.15 ± 0.16	3.54 ± 0.20	9.27 ± 0.07	7.75 ± 0.35	9.11 ± 0.20	5.38 ± 0.04
336	8.42 ± 0.10	8.90 ± 0.09	8.29 ± 0.08	3.50 ± 0.20	9.01 ± 0.14	7.97 ± 0.05	8.92 ± 0.28	4.74 ± 0.04
504	8.24 ± 0.12	8.63 ± 0.20	8.22 ± 0.02	3.51 ± 0.23	8.93 ± 0.27	8.06 ± 0.08	8.79 ± 0.04	3.03 ± 0.05
672	8.82 ± 0.13	8.90 ± 0.11	8.49 ± 0.44	3.25 ± 0.20	3.25 ± 0.20	8.09 ± 0.22	9.05 ± 0.17	1.72 ± 0.24

**Table 3:** The concentration (log CFU/mL) of lactic acid bacteria (LAB) and *L. monocytogenes* in mono-culture and co-culture set-ups using brain heart infusion broth and milk at 30 °C.

Time (h)	BHI				MILK			
	Mono-Culture		Co-Culture		Mono-Culture		Co-Culture	
	LAB	*L. monocytogenes*	LAB	*L. monocytogenes*	LAB	*L. monocytogenes*	LAB	*L. monocytogenes*
0	7.09 ± 0.03	4.32 ± 0.02	7.09 ± 0.07	4.10 ± 0.03	7.12 ± 0.03	4.30 ± 0.07	7.19 ± 0.05	4.32 ± 0.16
24	8.25 ± 0.08	4.32 ± 0.02	8.03 ± 0.08	4.10 ± 0.03	8.25 ± 0.04	4.30 ± 0.07	8.29 ± 0.04	4.32 ± 0.16
48	8.16 ± 0.06	7.18 ± 0.04	8.22 ± 0.02	3.46 ± 0.11	8.92 ± 0.03	7.31 ± 0.03	9.12 ± 0.13	3.60 ± 0.12
72	7.74 ± 0.18	8.10 ± 0.06	7.99 ± 0.08	3.35 ± 0.08	9.16 ± 0.05	8.20 ± 0.07	8.90 ± 0.13	0.00 ± 0.00
96	8.25 ± 0.08	8.01 ± 0.08	7.46 ± 0.08	2.88 ± 0.03	9.19 ± 0.13	8.05 ± 0.12	8.40 ± 0.09	0.00 ± 0.00
120	8.04 ± 0.04	8.76 ± 0.13	7.97 ± 0.04	1.30 ± 0.00	9.30 ± 0.07	8.20 ± 0.11	9.14 ± 0.18	0.00 ± 0.00

**Table 4:** Parameters of the primary and secondary models used for the description of bacterial mono-culture growth behaviour in BHI based on the one-step modelling approach.

Bacteria	Primary Models	*X*_0_	*X_max_*	T_0_	*b*_1_	*b*_2_	RMSE	*R* ^2^_adj_
LAB	Gompertz	7.17 ± 0.26	8.26 ± 0.22	1.88 ± 4.93	1.28 × 10^−2^ ± 1.26 × 10^−2^	0.49 ± 1.91	0.249	0.822
	Logistic	7.09 ± 0.31	8.23 ± 0.26	1.85 ± 3.46	1.50 × 10^−2^ ± 1.26 × 10^−2^	0.18 ± 1.68	0.276	0.781
	Baranyi	7.07 ± 0.64	8.26 ± 0.22	1.43 ± 4.40	1.14 × 10^−2^ ± 9.97 × 10^−3^	0.33 ± 2.46	0.250	0.820
	Huang	7.08 ± 0.32	8.27 ± 0.24	1.86 ± 3.82	1.86 × 10^−2^ ± 1.82 × 10^−2^	0.50 ± 6.34	0.264	0.800
*L. monocytogenes*	Gompertz	4.66 ± 0.51	8.63 ± 0.44	−20.01 ± 8.93	7.42 × 10^−3^ ± 3.49 × 10^−3^	4.00 ± 6.98	0.481	0.932
	Logistic	4.67 ± 0.50	8.63 ± 0.44	−18.29 ± 9.47	7.26 × 10^−3^ ± 3.19 × 10^−3^	3.02 ± 5.64	0.485	0.931
	Baranyi	4.59 ± 0.61	8.59 ± 0.40	−20.64 ± 8.52	7.30 × 10^−3^ ± 3.62 × 10^−3^	4.00 ± 7.30	0.482	0.931
	Huang	4.53 ± 0.65	8.58 ± 0.42	−20.73 ± 9.70	8.92 × 10^−3^ ± 3.95 × 10^−3^	4.00 ± 6.62	0.505	0.925

**Table 5:** Parameters of the primary and secondary models used for the description of bacterial mono-culture growth behaviour in MILK based on the one-step modelling approach.

Bacteria	Primary Models	*X*_0_	*X*_max_	T_0_	*b*_1_	*b*_2_	RMSE	*R* ^2^_adj_
LAB	Gompertz	7.08 ± 0.73	9.11 ± 0.24	−0.45 ± 2.12	9.40 × 10^−3^ ± 2.61 × 10^−3^	0.13 ± 1.39	0.229	0.925
	Logistic	7.18 ± 0.29	9.11 ± 0.23	−0.27 ± 1.84	1.13 × 10^−2^ ± 3.36 × 10^−3^	0.33 ± 0.75	0.230	0.925
	Baranyi	6.95 ± 1.12	9.10 ± 0.23	−0.77 ± 2.19	9.16 × 10^−3^ ± 2.56 × 10^−3^	0.09 ± 2.18	0.230	0.925
	Huang	7.13 ± 0.30	9.10 ± 0.22	−0.54 ± 2.18	1.43 × 10^−2^ ± 5.04 × 10^−3^	0.50 ± 3.48	0.230	0.925
*L. monocytogenes*	Gompertz	4.80 ± 0.40	7.96 ± 1.00	−8.41 ± 1.00	1.04 × 10^−2^ ± 4.87 × 10^−3^	4.00 ± 5.55	0.392	0.930
	Logistic	4.79 ± 0.43	7.96 ± 0.42	−10.41 ± 4.70	8.18 × 10^−3^ ± 3.59 × 10^−3^	2.46 ± 2.66	0.422	0.919
	Baranyi	4.75 ± 0.46	7.92 ± 0.36	−9.01 ± 3.87	1.00 × 10^−2^ ± 5.23 × 10^−3^	4.00 ± 6.81	0.408	0.924
	Huang	4.71 ± 0.51	7.98 ± 0.40	−9.14 ± 3.84	1.18 × 10^−2^ ± 4.20 × 10^−3^	4.00 ± 5.73	0.429	0.916

**Table 6:** Parameters and prediction capabilities of interaction models used for the description of bacterial co-culture growth behaviour in BHI and milk.

Medium	Temperature (°C)	Traditional Modelling Approach						Machine Learning Approach			
		Modified Jameson Model			Lotka–Volterra Model			RFR		GPR	
		*N_cri_*LAB (log CFU/mL)	RMSE	*R* ^2^_adj_	*F_LABLm_*	RMSE	*R* ^2^_adj_	RMSE	*R* ^2^_adj_	RMSE	*R* ^2^_adj_
BHI	4	7.69	0.368	0.972	1.13	0.324	0.978	0.437	0.960	0.242	0.988
	10	7.88			1.03						
	30	7.33			1.10						
milk	4	9.20	0.563	0.958	0.97	0.507	0.962	0.876	0.885	0.475	0.966
	10	8.00			1.08						
	30	6.57			1.31						

RMSE: Root mean square error calculated based on log CFU/mL. *R*^2^_adj_: Adjusted coefficient of determination.

## Data Availability

The data will be shared when any researchers request.

## Supplementary Materials

The following supporting information can be downloaded at: https://www.mdpi.com/article/10.3390/life15020244/s1, Figure S1: The illustration of developed software using Gaussian Process Regression.

## References

[1] Randhawa M.A., Asghar A., Nadeem M., Ahmad N. (2018). Food Safety: Benefits of Contamination Control on Consumers’ Health. Food Safety and Preservation.

[2] Kasra-Kermanshahi R., Mobarak-Qamsari E. (2015). Inhibition effect of lactic acid bacteria against food born pathogen, *Listeria monocytogenes*. Appl. Food Biotechnol..

[3] Costa JC C.P., Bover-Cid S., Bolívar A., Zurera G., Pérez-Rodríguez F. (2019). Modelling the interaction of the sakacin-producing *Lactobacillus sakei* CTC494 and *Listeria monocytogenes* in filleted gilthead sea bream (*Sparus aurata*) under modified atmosphere packaging at isothermal and non-isothermal conditions. Int. J. Food Microbiol..

[4] Favero-Longo S.E., Viles H.A. (2020). A review of the nature, role and control of lithobionts on stone cultural heritage: Weighing-up and managing biodeterioration and bioprotection. World J. Microbiol. Biotechnol..

[5] Şanlıbaba P., Buzrul S. (2022). Control of *Listeria monocytogenes* in milk by using phage cocktail. Sci. Agropecu..

[6] Barache N., Belguesmia Y., Martinez B., Seal B.S., Drider D. (2024). Bacteriocins and Bacteriophages as Dual Biological Players for Food Safety Applications. Encyclopedia.

[7] Herody C., Soyeux Y., Hansen E.B., Gillies K. (2010). The legal status of microbial food cultures in the European Union: An overview. Eur. Food Feed. Law Rev..

[8] Bourdichon F., Casaregola S., Farrokh C., Frisvad J.C., Gerds M.L., Hammes W.P., Harnett J., Huys G., Laulund S., Ouwehand A. (2012). Food fermentations: Microorganisms with technological beneficial use. Int. J. Food Microbiol..

[9] Melian C., Ploper D., Chehín R., Vignolo G., Castellano P. (2024). Impairment of *Listeria monocytogenes* biofilm developed on industrial surfaces by *Latilactobacillus curvatus* CRL1579 bacteriocin. Food Microbiol..

[10] Khalid K. (2011). An overview of lactic acid bacteria. Int. J. Biosci..

[11] Mejlholm O., Dalgaard P. (2015). Modelling and predicting the simultaneous growth of *Listeria monocytogenes* and psychrotolerant lactic acid bacteria in processed seafood and mayonnaise-based seafood salads. Food Microbiol..

[12] Quinto E.J., Marín J.M., Schaffner D.W. (2016). Effect of the competitive growth of *Lactobacillus sakei* MN on the growth kinetics of *Listeria monocytogenes* Scott A in model meat gravy. Food Control.

[13] Costa J.C.C.P., Bolívar A., Pérez-Rodríguez F. (2023). Mathematical Simulation of the Bio-Protective Effect of Lactic Acid Bacteria on Foodborne Pathogens. Basic Protocols in Predictive Food Microbiology.

[14] Bintsis T. (2018). Lactic acid bacteria: Their applications in foods. J. Bacteriol. Mycol..

[15] FDA (Food and Drug Administration) (2018). GRAS Notice Inventory. USA. https://www.fda.gov/food/generally-recognized-safe-gras/gras-notice-inventory.

[16] Koutsoumanis K., Allende A., Álvarez-Ordóñez A., Bolton D., Bover-Cid S., Chemaly M., Davies R., Hilbert F., Lindqvist R., EFSA Panel on Biological Hazards (BIOHAZ) (2019). Update of the list of QPS-recommended biological agents intentionally added to food or feed as notified to EFSA 9: Suitability of taxonomic units notified to EFSA until September 2018. Efsa J..

[17] Pérez-Rodríguez F., Valero A. (2013). Predictive Microbiology in Foods.

[18] Tarlak F. (2023). The Use of Predictive Microbiology for the Prediction of the Shelf Life of Food Products. Foods.

[19] Qian X., Tian P., Zhao J., Zhang H., Wang G., Chen W. (2022). Quorum Sensing of Lactic Acid Bacteria: Progress and Insights. Food Rev. Int..

[20] Iulietto M.F., Sechi P., Cella E., Grispoldi L., Ceccarelli M., Al Ani A.R., Işıklar B., Anil H.M., Cenci-Goga B.T. (2018). Inhibition of *Listeria monocytogenes* by a formulation of selected dairy starter cultures and probiotics in an in vitro model. Ital. J. Anim. Sci..

[21] Zwietering M.H., Jongenburger I., Rombouts F.M., Van’t Riet K.J. (1990). Modeling of the bacterial growth curve. Appl. Environ. Microbiol..

[22] Baranyi J., Roberts T.A. (1994). A dynamic approach to predicting bacterial growth in food. Int. J. Food Microbiol..

[23] Huang L. (2017). IPMP Global Fit–A one-step direct data analysis tool for predictive microbiology. Int. J. Food Microbiol..

[24] Juneja V.K., Melendres M.V., Huang L., Gumudavelli V., Subbiah J., Thippareddi H. (2007). Modeling the effect of temperature on growth of *Salmonella* in chicken. Food Microbiol..

[25] Ratkowsky D.A., Olley J., McMeekin T.A., Ball A. (1982). Relationship between temperature and growth rate of bacterial cultures. J. Bacteriol..

[26] Jameson J.E. (1962). A discussion of the dynamics of *Salmonella* enrichment. Epidemiol. Infect..

[27] Le Marc Y., Valík L., Medveďová A. (2009). Modelling the effect of the starter culture on the growth of *Staphylococcus aureus* in milk. Int. J. Food Microbiol..

[28] Bolívar A., Tarlak F., Costa J.C.C.P., Cejudo-Gómez M., Bover-Cid S., Zurera G., Pérez-Rodríguez F. (2021). A new expanded modelling approach for investigating the bioprotective capacity of *Latilactobacillus sakei* CTC494 against *Listeria monocytogenes* in ready-to-eat fish products. Food Res. Int..

[29] Cornu M., Billoir E., Bergis H., Beaufort A., Zuliani V. (2011). Modeling microbial competition in food: Application to the behavior of *Listeria monocytogenes* and lactic acid flora in pork meat products. Food Microbiol..

[30] Costa JC C.P., Bolívar A., Valero A., Carrasco E., Zurera G., Pérez-Rodríguez F. (2020). Evaluation of the effect of *Lactobacillus sakei strain* L115 on *Listeria monocytogenes* at different conditions of temperature by using predictive interaction models. Food Res. Int..

[31] Yücel Ö., Tarlak F. (2023). An intelligent based prediction of microbial behaviour in beef. Food Control.

[32] Coelho M., Silva C., Ribeiro S., Dapkevicius M., Rosa H. (2014). Control of *Listeria monocytogenes* in fresh cheese using protective lactic acid bacteria. Int. J. Food Microbiol..

[33] Nielsen D.S., Cho G.S., Hanak A., Huch M., Franz C.M., Arneborg N. (2010). The effect of bacteriocin-producing Lactobacillus plantarum strains on the intracellular pH of sessile and planktonic *Listeria monocytogenes* single cells. Int. J. Food Microbiol..

