# Supplementary material for: The Development of Machine Learning-Assisted Software for Predicting the Interaction Behaviours of Lactic Acid Bacteria and Listeria monocytogenes
Source: Life (Basel). 2025 Feb 6;15(2):244. doi: 10.3390/life15020244 (PMC11856248; doi:10.3390/life15020244)
Supplement: Supplementary file 1 [file life-15-00244-s001.zip › life-3408079-supplementary.pptx]

## Slide 1
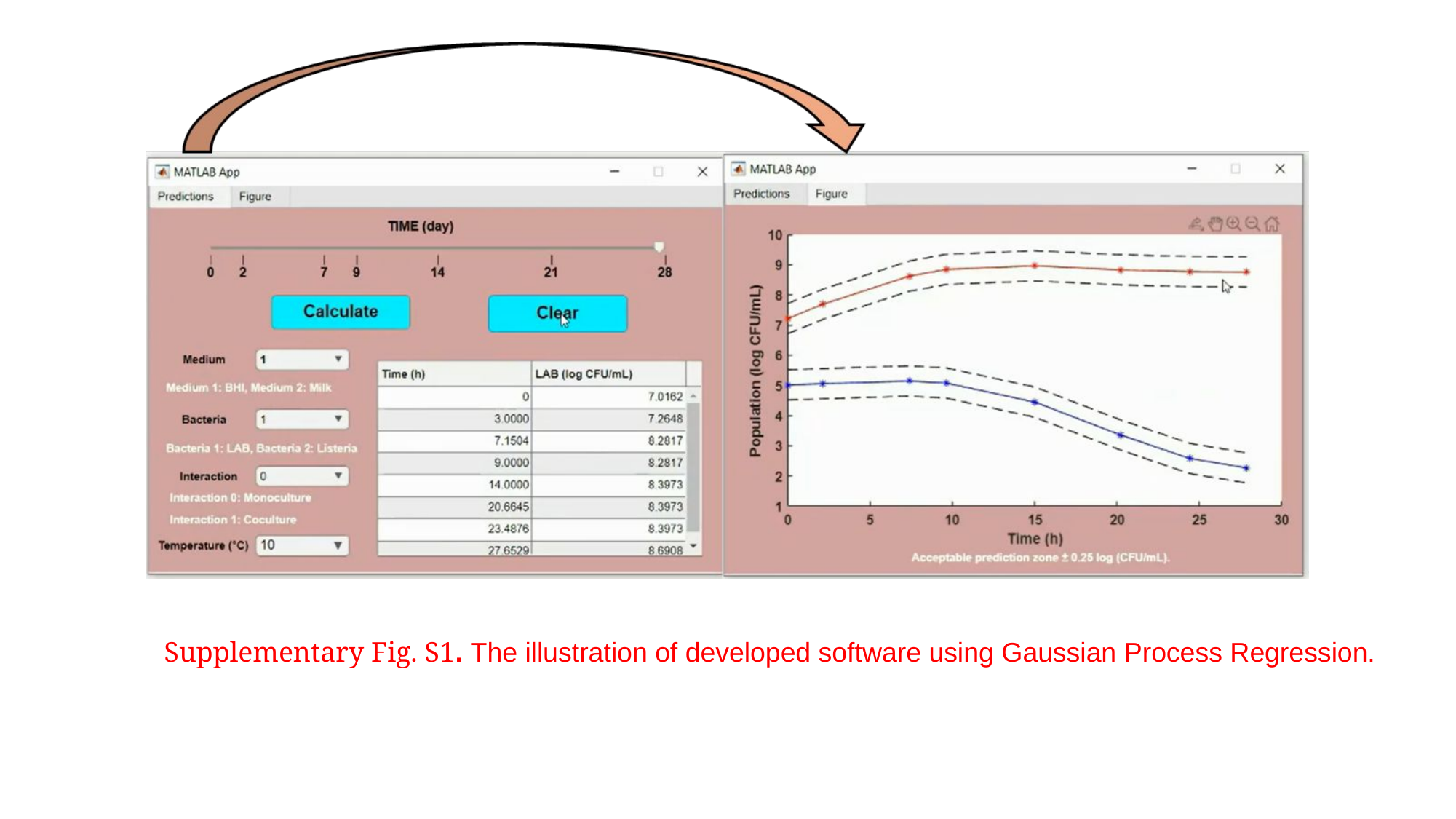

Supplementary Fig. S1. The illustration of developed software using Gaussian Process Regression.
